# Statistical science: a grammar for research

**DOI:** 10.1007/s10654-017-0288-1

**Published:** 2017-07-29

**Authors:** David. R. Cox

**Affiliations:** Nuffield College, Oxford, Oxford, OX1 1NF UK

**Keywords:** Design of studies, Metrology, Interpretation, Causality, Uncertainty

## Abstract

I greatly appreciate the invitation to give this lecture with its century long history. The title is a warning that the lecture is rather discursive and not highly focused and technical. The theme is simple. That statistical thinking provides a unifying set of general ideas and specific methods relevant whenever appreciable natural variation is present. To be most fruitful these ideas should merge seamlessly with subject-matter considerations. By contrast, there is sometimes a temptation to regard formal statistical analysis as a ritual to be added after the serious work has been done, a ritual to satisfy convention, referees, and regulatory agencies. I want implicitly to refute that idea.

## Introduction

I greatly appreciate the invitation to give this lecture with its century long history. The title is a warning that the lecture is rather discursive and not highly focused and technical. The theme is simple. That statistical thinking provides a unifying set of general ideas and specific methods relevant whenever appreciable natural variation is present. To be most fruitful these ideas should merge seamlessly with subject-matter considerations. By contrast, there is sometimes a temptation to regard formal statistical analysis as a ritual to be added after the serious work has been done, a ritual to satisfy convention, referees, and regulatory agencies. I want implicitly to refute that idea.

Statistical notions aim to provide a reasonably coherent way of thinking about variability and its impact, various specialized techniques of study design and analysis and, in particular, a way of quantifying the security of ones conclusions. That last aspect, the whole elaborate apparatus of significance tests, confidence distributions, posterior distributions and so on quite often is overemphasized, but is nevertheless clearly important and intellectually challenging.

## A sequence

It is helpful to think about these issues in the following sequence: the order is not to be taken too seriously

- Question formulation
- Choice of study individuals
- Study design
- Metrology
- Data collection and monitoring, that is quality control
- Data analysis, through various stages
- Presentation of conclusions
- Interpretation, including decision implications, possibly about the welfare of patients, policy implementation, and possibly about what to do next

The bulk of the formal statistical literature is on analysis, probably too much of it, as I have already said, on the formal assessment of security.

## Question formulation

There is much to be said about all the themes I have listed above but I am going to make only some isolated comments, including a few historical points.

In all fields of work, even in pure mathematics, the formulation of issues or questions for investigation is central. Better a rough answer to an important issue than a beautiful study of a topic of no real concern. Statistical considerations enter in at least two ways. The first is to ensure that the questions are reasonably defined and capable of being addressed. Then, do we have or can we collect data capable of giving a reasonable answer?

Then, secondly, and more specifically, there is the so-called factorial principle. In many, but not all, contexts, experimental and observational, it is wise to ask a series of interrelated questions rather than a single such question.

There are already important issues implicit.

In a few fields such as infectious disease epidemiology initial investigation may be helped by simple, if highly idealized mathematical models. For example in a fairly recent veterinary study of bovine TB the special issue arose of the interaction between the disease in two species, cattle and wildlife, each affected its own and the other. The effect on cattle of intervening on the wild life was studied mathematically and gave some partial guidance to a 10 years experimental study of the intervention.

In initial investigation of a broad topic simplicity may be central.

## A little history

On the theme of simplicity let me digress into a piece of history. In 1930 a very early nutrition study, the Lanarkshire milk experiment, was set up in a very poor area of Scotland. Within each of a considerable number of classes of children, individuals were essentially randomized between free milk each day and control. In some schools the milk was pasteurized and in some not. The design was such that in each class of children taking part the heights and weights of the children were measured and the children divided into two groups, either by an essentially random device or by alternation down the alphabetical list of the childrens’ surnames. Then the teachers had the opportunity to “improve” the assignment. About 30,000 children were involved. After 3 months the children were remeasured.

Although he was not involved in the experiment the statistician Student [8] published a gently worded but powerful critique of the study showing, in particular, that altering the randomization in what was in fact a biased way had made the experiment virtually uninterpretable. There were major issues involved also in the measurement process. Student also suggested that as a preliminary study in what was an open field a matched pair investigation using twins might have been preferable. He did point out though that the design would be sensitive to mischievous behaviour by the twins as well as to a broader criticism that the response pattern of twins might be atypical.

And that gives me the excuse to say something about Student.

He was educated as a chemist and had in his undergraduate degree attended an introductory course in calculus, etc. He spent his whole career working for Guiness’s, the brewers based in Dublin. He became interested in statistical issues through the lab work in Dublin and from their concern with agricultural trials of barley. His name was W.S. Gosset but to protect the interests of his firm employees were not allowed to publish under their own name.

He published a fairly small number of statistical papers several, that on the Student *t* test for example, of very high originality and lucidity.

His work illustrates, in particular, the point that those with very limited mathematics can make massive contributions to statistical thinking, in particular by asking focused questions. Many mathematicians in the 50 years before Student’s very clumsy derivation of the *t* distribution could have derived it far more elegantly, but they did not pose the right question. He is far from the only example of this phenomenon.

One moral of the Lanarkshire experiment may be that while planned complexity can be very fruitful in many contexts this may not be so when there is little history of previous investigation.

## A modern emphasis

The modern emphasis is on large-scale studies of which very notable examples are the Rotterdam study, the Women’s Health Initiative and the Million Women study and a number of impressive Chinese large-scale wide-ranging investigations. There is literature on the design of such observational studies but there is some possibly unexplored parallel with the old statistical literature on the design of factorial experiments that was developed in an agricultural context in the 1920’s and 1930’s and elaborated in different form in an industrial context 30–40 years later. There may be scope for some of these ideas in the context of large observational studies focusing perhaps on measuring key features on all individuals but being more selective in measuring less central features.

## Metrology

I pass rapidly through some other phases, metrology for example: in many contexts a key to progress is the ability to measure features in a reasonably convenient and accurate way. Thus, how should one assess health-related quality of life? One of my colleagues comparing two regimes for dealing with arthritis asked at each clinic visit: did you put your socks and shoes on unaided today? The question was not intrusive and likely to receive a reasonably truthful answer. In other contexts, one single score (QALY) may be appropriate, much loved by health economists, no doubt at least in part because of the one-dimensional nature of so much theoretical thinking in economics, forced by the tyranny of wanting to optimize something. A richer assessment will involve a lengthy questionnaire with its attendant difficulties in implementation.

How reliable are the conclusions drawn from various scanning devices, and so on? How reproducible are the images and how firmly based is their interpretation? These are typical issues of metrology and their careful investigation calls for appropriate statistical design and analysis.

## Study design

In connection with study design let me comment first about randomization.

It is common to contrast observational studies with randomized clinical trials, with implicit or often explicit emphasis on the word *randomized.* This is sometimes misleading. The contrast is often better of observational studies with studies in which treatment allocation is by a defined algorithm specified by the investigator. In an observational study we may observe meticulously that individual A received exposure or treatment T but have no direct knowledge of exactly how or why that came about; in an intervention or experiment we do know exactly why. Because a well-specified rule has dictated so.

Now in some cases it is crucial that the assignment involves randomization and in many situations randomization is certainly desirable. Moreover often one element of randomization may be far from enough. On the other hand, just occasionally it is a bad idea if allocation involves randomization.

How can that come about? In two ways.

Here is a real example, admit**t**edly not a biomedical one. In a particular laboratory-based study, each section of a large investigation involved a set of 8 specimens each of which needed a rather different procedure taking about 5 min per specimen of dedicated work in the lab. There were many such sets of 8, each thus involving about 40 min intensive work in appropriate sequence. Now if the order of each of the many sets of 8 were to have been independently randomized there would have been increased possibility of sets getting the “wrong” treatment and of this being undetected. It was decided to take the specimens in systematic order, occasionally altered, and to have careful quality control checks of the lab work, by insertion blind of occasional “dummy” sets of specimens. Separate randomization of each set of eight would have been a bad idea.

A general and different point is that, powerful and protective though randomization is, just because a study involves one randomization may be far from enough. Systematic distortion may enter at many phases of an investigation.

A second quite different possibility is that a very small number of units may be involved. An example would be a trial of a community intervention policy using four communities, two “treated” and two “control”. Here the very few possible arrangements all have distinctive features and it would be wise to choose the one with fewest adverse features, or the features most easily adjusted by analysis, possibly randomizing the names. One important conceptual and theoretical requirement for randomization is that all, or virtually all, the arrangements in the randomization set are equally effective. It is said that R.A. Fisher, [7] who introduced formal randomization into experimental design, was once asked: what should be done if the randomization throws up a clearly undesirable allocation? Fisher is said to have reacted with surprise: rerandomize of course. It is important that this is both the practical and theoretically correct procedure.

## Analysis

The Princeton and Bell Labs statistician J.W. Tukey emphasized what he called exploratory data analysis, essentially descriptive statistics, and was surely right to stress the underemphasized importance of such ideas, although they should be linked with the more formal side as far as possible.

I want to consider briefly three levels of approach to a standard type of study of dependence.

Focus first on the simplest situation. How does an outcome (blood pressure, quality of life, survival, for example) depend on explanatory variables, whether they are intrinsic properties of the patients or exposures or treatments? We can distinguish many kinds of such study but for the moment I want to concentrate on just three, a very crude classification;

We may have almost a text-book situation. There may be a modest number of explanatory variables and an appreciable but not enormous number of patients. So depending on the nature of the outcome, continuous measurement, binary response or survival time, say, we fit a standard-type of model. Subject to checks for outliers, nonlinearities, interaction terms, possible anomalous groups of observations and other peculiarities the conclusion may be clear and in some cases a formal analysis may hardly be needed, a simple graphical or tabular summary being totally adequate. Of course planning a study must embrace planning an analysis, in particular to ensure that the study is in principle capable of answering the questions of interest. But perhaps especially if a long time elapses between the initial phases and the analysis of the data total adherence to the initial analysis may for a range of reasons be wrong.

The second possibility is that there are a large number of explanatory variables and an even larger number of individuals. I am not going to discuss the literature on backwards selection, forward selection and combinations thereof and the role of penalized methods of optimization such as ridge regression in which the measure of lack of fit, for example a sum of squares of residuals is supplemented by a multiple of the sum of squares of the regression coefficients in suitably standardized form. This has the effect of shrinking the regression coefficients selectively towards zero.

Instead I want to go to the third case of an enormous number of explanatory variables and a limited number of study individuals, such as arise in particular in genomics; lets say we have of the order of 100 individuals and 10,000 or more explanatory variables. Tibshirani [9] suggested the lasso in which, in particular, the penalty for small regression coefficients was relatively greater forcing many of them to zero and thus achieving a simple sparse model with adequate fit. That produces a single model. That may be fine for empirical prediction but for interpretation it is not satisfactory if there are many different choices that give imperceptively different fits. We would like in some sense a confidence set of those simple models that give adequate fit.

I want to outline some recent work [5] with Dr Heather Battey, Dept of Mathematics, Imperial College London, which aims at this.

Our example from the genomics literature had rather over 100 patients for whom there was an outcome and for whom microarrays contained probes for tens of thousands of genes.

Clearly there must be some notion of sparsity of effects. How should that be used?

In general we arrange the variables in a roughly 10 × 10 × 10 cube if there are of the order of 10^3^ variables and in a 10 × 10 × 10 × 10 hypercube if there are of the order of 10^4^ variables. Do a large number of standard regression analyses regressing outcome on the sets of say 10 variables formed from rows, columns, edges, etc. Thus with 10^3^ variables in a cube there are 300 standard regressions in which each variable occurs three times always in conjunction with different companions.

From each of these small regressions we select say the two most significant variables. We repeat until a relatively small number of relatively simple potential explanations remain.

The essence is that if there are alternative well-fitting explanations of the data we should aim to specify them not choose one somewhat arbitrarily.

A general point is that the motivation for this procedure comes directly from the work 80 years ago by F. Yates on plant breeding studies where say 1000 varieties of wheat were studied in simple block designs and gradually over a few years reduced to a small number for agricultural use. For this Yates introduced and studied so-called partially balanced incomplete block designs. These are being used here. This is illustrates in an extreme case the transference of methodological ideas across fields.

The essence of Dr Battey’s and my formulation is that we are not predicting but attempting to suggest explanations, recognizing that there may be many explanations that fit about equally well and that further expert information or additional experimentation will be needed to resolve the interpretation. The problem is essentially exploratory and to force a single answer when there are several or many virtually equally well-fitting but different fits is misleading.

I have deliberately described the procedure in an informal way. It does have some formal theoretical statistical properties, however, derived under highly idealized conditions. That is not the central point, though.

## Interpretation

The final and in some ways most challenging phase is interpretation. Suppose we have some reasonably secure conclusions. What are their real implications?

For example, consider a randomized comparison of a treatment, T, with control, C, under the unrealistic assumptions that there is complete adherence, and that the patients can be treated as a random sample from the relevant population. Suppose that there is a clear superiority of T, highly significant statistically. What does this show?

Does it mean that T is better for every patient? Obviously not. What it shows formally is something like the following.

Consider two hypothetical situations. First, all individuals receive T, all else being unchanged. Secondly, and notionally quite separately, all individuals receive C all else being unchanged. Then there is strong evidence that the aggregate of all outcomes under T is better than the aggregate outcome under C. This does not preclude there being a minority of individuals for whom T is bad news.

Will the conclusion generalize to a new population, inevitably somewhat different from the context of the current investigation?

The problem is also acute for what is sometimes called the issue of specificity. A treating clinician has a particular patient about whom she or he has fairly detailed information. Should he or she recommend T or should the recommendation be C?

So we have two big problems: generalizability and specificity.

## Some more history

So far the word causality has not been mentioned. But first there is some more history.

W.G. Cochran was a Scot educated in mathematics and physics at University of Glasgow and continuing to Cambridge, UK, to do postgraduate work in fluid dynamics but switching to statistics, then in a lively phase under the direct and indirect influence of R.A. Fisher, and moved to agricultural research at Rothamsted to work with Yates, Fisher’s successor there. Cochran published important papers in that period, in particular one with Yates on what was in effect the first systematic study of metaanalysis or overviews. Cochran emigrated and in 1957 moved to Harvard where he became of course a pivotal figure in the US and internationally.

In 1965 Cochran [2] returned to UK on study leave and during that time read to the RSS a paper arguing that the time was right for developing ideas for the interpretation of observational studies to parallel the work on interventions, that is experiments. The vote of thanks was proposed by Bradford Hill who set out his considerations or guidelines pointing towards causality; he strongly emphasized these were not to be regarded as either necessary or as sufficient conditions; see Bradford Hill [1].

It is hard to trace all influences but I suspect that the direct and indirect influence of Cochran’s work has been pivotal, nationally and internationally.

On a personal note, I met Cochran only a couple of times briefly, but well before that, before he moved to Harvard, he had been exceptionally encouraging and helpful to me in correspondence about issues of experimental design and I have always been very grateful for that.

His 1965 paper was, in particular, surely one of the starts of the current interest in causality.

## Causality

There are broadly at least three views of causality in the literature; for a brief review, see Cox and Wermuth [6].

First, largely in the time series field, there is Wiener-Granger causality essentially about the ability of one time series to predict the future of another. Wiener was an outstanding MIT pure mathematician and Granger an econometrician.

The second and widely used definition involves the notion of an exposure being hypothetically changed, other things being equal. It can be regarded as underpinning the classical theory of randomized experiments and, generalized into broader settings, it has a large and rich literature.

The third notion adds to the second some notion of evidence-based explanation in terms of an underlying process, biological or physical perhaps. Of course such explanations are not “ultimate”. Their danger is that they can nearly always be manufactured after the event, but very much more than that is required, typically explicit independent evidence. Davey-Smith coined the term triangulation for this view of causality.

For example, in the veterinary study I mentioned briefly above, an unexpected conclusion seemed to emerge, that a control policy that was intended to reduce incidence of TB actually increased incidence. There were two possible explanations. A subsidiary experiment showed that one of the explanations, that the control procedure led to infected individuals, although fewer in number, travelling more widely and hence producing more infection, was consistent with the data.

This brings us back to a key question: what justifies generalization and specificity?

Random sampling: not really or at least very rarely!

Overall outcome of randomized trial or observational near equivalent: to a limited extent.

Stability of such an effect with respect to key intrinsic features, not to be confused with subgroup analysis. Certainly this is important. Although only a limited number of such checks can be made in any one context, the importance of assessing stability probably deserves more emphasis in some current contexts.

The above supplemented by third level causality, the last often a relatively more fragile route.

Of course the smaller the effect the more delicate the arguments that are needed, one of Bradford Hill’s points.

I have touched on just a few of the many broad issues that can reasonably be called statistical and repeat that integration into the subject-matter thinking of the field in question is crucial for success: it not an issue of imposing some ritualistic procedures of analysis or design.

The bulk of the formal statistical literature is on analysis, probably too much of it on the formal assessment of security by significance tests, confidence distributions and posterior distributions with the mass of challenging conceptual issues that they raise.

What is the main limitation of the kind of thinking I am characterizing as statistical? It is probably the emphasis, touched on at the end of my remarks on causality, on the self-contained security of individual investigations and of series of investigations that can be formulated as estimating a common feature. For example, even though the studies of Bradford Hill and Doll among others had by the early 1950’s shown compelling evidence of the harmful effect of tobacco smoking, there was among some senior statisticians, including some not well known for agreeing with one another, widespread scepticism about the causal nature of the effect. Cornfield et al. [3] showed in a powerful paper that when various different kinds of evidence were assembled together a virtually overwhelming case emerged; an illustration of the general point made earlier about synthesizing different kinds of evidence. It is hard to see a general quantitative basis for such wide-ranging synthesis of evidence, although for an individual research worker a subjectivist view of probability might be invoked.

## Some broad issues

First how should statistical ideas be taught?

If there is a general principle involved in teaching it may be: Approach the new and unknown out of the known! So for a mathematician one would emphasize the axioms of probability theory and their links with other areas of mathematics: for a research worker in a specific field one would start with a possibly idealized example of an important issue in that field. For a very broad audience one might start with topics from every-day life, and so on. In so far as feasible, emphasize principle over specific technique but in so doing for most types of audience specific examples are crucial.

There is what might broadly be called an ethical issue for statisticians, especially those in medical and associated fields. Research is not for the timid and negative thinking is dangerous. But statisticians are quite often in the position of having to sound warnings against overinterpretation arising from poor design and or inadequate scale. The empirical evidence is overwhelming that such warnings are necessary; our newspapers are almost daily describing studies often with claims of strong implications for our well-being, claims with at best dubious base. The answer is to emphasize the role of general methodological considerations in the many well-designed important studies that do contribute so much to human welfare.

## The future

We live in a dynamic age. There is Big data; Machine learning; Data science; Deep learning. And the list is probably already obsolete. There are surely important ideas and problems in all of them, largely based on the computer technology involved.

Big data have been around all my working life and no doubt much longer, but of course in earlier times had to be analysed on a sampling basis. Key issues are their quality and relevance. Over quality, some big data, such as that coming from CERN in connection with the discovery of the Higgs boson, is undoubtedly of very high quality. But quality may be less clear in other contexts. If a little bad data is a little bit misleading what are the consequences of a large amount of bad data? Next, are the study individuals, especially if self-selected, appropriate for the issue under investigation? Finally there is a more subtle statistical issue of precision. Estimates of precision, however calculated, often involve explicit or implicit assumptions of the statistical independence of individuals, leading to standard errors of key estimates that implicitly are inversely proportional to the square root of the number of individuals involved and hence very small. Such estimates of error indeed are often so small as to be intrinsically implausible and theoretical arguments can be produced to show why this is [4].

More broadly and importantly the emphasis of the recently popular themes is primarily on empirical prediction. Important though that is, a deeper and ultimately more probing approach is required for understanding, and ultimately also for stable prediction beyond the immediate environment.

That is, the challenge in part is to give all these new ideas a broader and richer perspective.

I greatly appreciate the invitation to give the Cutter Lecture. The paper is a slightly extended version of that Lecture. I thank Bianca de Stavola and Heather Battey for helpful comments.
